# High-throughput sequencing identification of novel and conserved miRNAs in the *Brassica oleracea* leaves

**DOI:** 10.1186/1471-2164-14-801

**Published:** 2013-11-19

**Authors:** Anna Lukasik, Halina Pietrykowska, Leszek Paczek, Zofia Szweykowska-Kulinska, Piotr Zielenkiewicz

**Affiliations:** 1 Institute of Biophysics and Biochemistry, Polish Academy of Sciences, Pawinskiego 5a, 02-106 Warsaw, Poland; 2 Department of Gene Expression, Institute of Molecular Biology and Biotechnology, Faculty of Biology, Adam Mickiewicz University, Umultowska 89, 61-614 Poznan, Poland; 3 Department of Immunology, Transplant Medicine and Internal Medicine, Medical University of Warsaw, Nowogrodzka 59, 02-006 Warsaw, Poland; 4 Department of Plant Molecular Biology, Institute of Experimental Plant Biology and Biotechnology, University of Warsaw, Miecznikowa 1, Warsaw, 02-096 Poland

**Keywords:** miRNA, *Brassica oleracea*, Cabbage, Target gene, High-throughput sequencing

## Abstract

**Background:**

Plant microRNAs are short (~21 nt) non-coding molecules that regulate gene expression by targeting the mRNA cleavage or protein translation inhibition. In this manner, they play many important roles in the cells of living organisms. One of the plant species in which the entire set of miRNAs has not been yet completely identified is *Brassica oleracea* var. *capitata* (cabbage). For this reason and for the economic and nutritional importance of this food crop, high-throughput small RNAs sequencing has been performed to discover the novel and conserved miRNAs in mature cabbage leaves.

**Results:**

In this study, raw reads generated from three small RNA libraries were bioinformatically processed and further analyzed to select sequences homologous to known *B. oleracea* and other plant miRNAs. As a result of this analysis, 261 conserved miRNAs (belonging to 62 families) have been discovered. MIR169, MIR167 and MIR166 were the largest miRNA families, while the highest abundance molecules were miR167, miR166, miR168c and miR157a. Among the generated sequencing reads, miRNAs* were also found, such as the miR162c*, miR160a* and miR157a*. The unannotated tags were used in the prediction and evaluation of novel miRNAs, which resulted in the 26 potential miRNAs proposal. The expressions of 13 selected miRNAs were analyzed by northern blot hybridization. The target prediction and annotation for identified miRNAs were performed, according to which discovered molecules may target mRNAs encoding several potential proteins – e.g., transcription factors, polypeptides that regulate hormone stimuli and abiotic stress response, and molecules participating in transport and cell communication. Additionally, KEGG maps analysis suggested that the miRNAs in cabbage are involved in important processing pathways, including glycolysis, glycerolipid metabolism, flavonoid biosynthesis and oxidative phosphorylation.

**Conclusions:**

Conclusively, for the first time, the large set of miRNAs was identified in mature cabbage leaves. Potential targets designation for these miRNAs may suggest their essential role in many plants primary biological processes. Presented study not only supplements the knowledge about *B. oleracea* miRNAs, but additionally it may be used in other research concerning the improvement of the cabbage cultivation.

## Background

Plant microRNAs (miRNAs) are a class of small, single-stranded RNAs that regulate gene expression by promoting cleavage or translation inhibition of the cognate mRNAs [1]. The majority of identified miRNAs have been shown to be evolutionarily conserved among a wide variety of plant species [2-4]. These short (18–24 nt) miRNA molecules are formed in a multistep process that takes place in the nucleus and involves several specific proteins. MicroRNA genes are transcribed by the RNA Polymerase II. Long primary transcripts (pri-miRNAs), with miRNA and miRNA* in the stem of hairpin structure, are processed by a multi-protein complex into the shorter precursor forms containing stem and loop structure (pre-miRNAs) [5,6]. The conversion of pri-miRNA to the pre-miRNA requires Dicer Like 1 (DCL1) RNase activity. The DCL1 interacts with the Hyponastic Leaves 1 (HYL1/DRB1) protein, binding the double stranded RNA [7,8]. Furthermore, the DCL1 acts with the Serrate (SE) protein that in turn binds to Cap Binding Complex (CBC), formed of the Cap Binding Protein 20 (CBP 20) and the Cap Binding Protein 80 (CBP 80/ABH 1) [9]. The Dawdle (DDL) protein stabilizes the pri-miRNA and facilitates its conversion to the pre-miRNA [10]. Recent studies have shown that additional proteins are also essential in the pri-miRNA processing, specifically the TOUGH (TGH) protein which interact with the DCL1 and HYL1 protein [11], and the SICLE (SIC) protein acting with HYL1 protein [12]. It is also known that C-terminal domain phosphatase-like 1 (CPL1) protein is necessary for the HYL1 dephosphorylation and conditioning its role in the miRNA biogenesis [13]. Additionally, Stabilized 1 (STA1), an *Arabidopsis* pre-mRNA processing factor 6 homolog, is a new potential molecule involved in miRNA biogenesis [14,15]. Pre-miRNA is further cleaved by DCL1 to a double stranded RNA formed by miRNA and miRNA*. This duplex contains two nucleotide overhangs at their 3′ ends, which are further methylated by HUA Enhancer 1 (HEN1) methyltransferase [16,17]. Methylated dsRNA is exported from the nucleus to the cytoplasm by HASTY (HST-1) exportin, an ortholog of exportin 5 in animals [18,19]. In the cytoplasm, miRNA:miRNA* duplex is loaded on the RISC (RNA Induced Silencing Complex) and then miRNA* is degraded [20]. The miRNA-guided RISC binds to target mRNA and directs its cleavage or translation inhibition [5], resulting in the down-regulation of this targeted gene expression. In this manner, miRNAs control crucial processes like plant development, organ formation, flowering timing and nutrient homeostasis. They also regulate response to the oxidative and salt stress, water deficit, cold, UV radiation and many biotic stresses [21].

Due to the important functions of the miRNAs and the recent advances of experimental and computational analytical approaches, interest in these small molecules has increased significantly in recent years. To date, there are 25141 mature miRNA sequences from 193 different species (ranging from viruses to human) collected in the miRBase database (release 19.0, August 2012) [22]. In contrast, the Plant MicroRNA Database (PMRD, release of June 11, 2012) [23] contains 10597 miRNAs identified in 127 plants. A great contributor to the amount of recently discovered miRNAs has been the development of high-throughput sequencing methods such as the Roche 454 Life Sciences System, Illumina Genome Analyzer and Applied Biosystems SOLiD system [24]. These technologies have been used in many studies to identify and determine the expression levels of miRNAs that are, for example, conserved, novel, tissue- or developmental stage-specific [25-29]. Experimental miRNA analyses are often supplemented by bioinformatic methods, which are used to process raw sequencing data, predict miRNA genes, precursors, mature sequences and targets, identify isoforms, and classify small RNAs into known miRNA families [24,30,31]. These experimental and computational methods not only allow for low-cost quantitative and qualitative small RNAs analysis but also generate more specific results in shorter time frame [32].

Among food crops, *Brassica oleracea* L. is an important and large group of vegetable subspecies, which include the cabbage, broccoli, Brussels sprout, kohlrabi, cauliflower, and kale. These plants have gained a special place in the human diet because of their nutritional benefits and the fact that as a food reserves can be stored in winter. Cabbage, as an example, is a great source of fiber, vitamin C, carotenoids, minerals, lupeol and glucosinolates, which have been shown to possess anti-inflammatory and anti-carcinogenic properties [33,34]. The *B. oleracea* crops are diploids that belong to the *Brassica* genus. Their CC genome, containing two copies of 9 chromosomes (2n = 2 x = 18), is still incomplete. To date, there are 202 potential *B. oleracea* miRNA candidates, the majority of which were obtained from a comparative genome-based computational analysis [35] or BLAST search [36]. Given the economic and nutritional importance of these vegetables in many countries, the identification and validation of all miRNAs in one of the *B. oleracea* subspecies – cabbage, is extremely important for biological-cognitive, agricultural and health reasons.

In the present study, for the first time, small RNA sequencing using the Illumina method was performed to identify novel and conserved miRNAs in cabbage leaves. A total of 287 miRNAs were identified, including 261 molecules that are conserved among plants and 26 that are novel candidates. The expression levels of 13 miRNAs were validated by northern blot hybridization. Furthermore, the best potential targets for all *B. oleracea* miRNAs were collected. In addition, the *trans*-acting siRNAs prediction was performed, which resulted in proposal of 203 tasiRNAs from 27 TAS loci, including the identified *A. thaliana* TAS3a homologue. To determine the functions of the discovered miRNAs and the processes which they potentially regulate in cabbage leaves, the GO annotation, GO enrichment analysis and KEGG (Kyoto Encyclopedia of Genes and Genomes) pathway mapping of predicted targets were performed.

## Results

### Analysis of the small RNA tags

**Figure 1:**
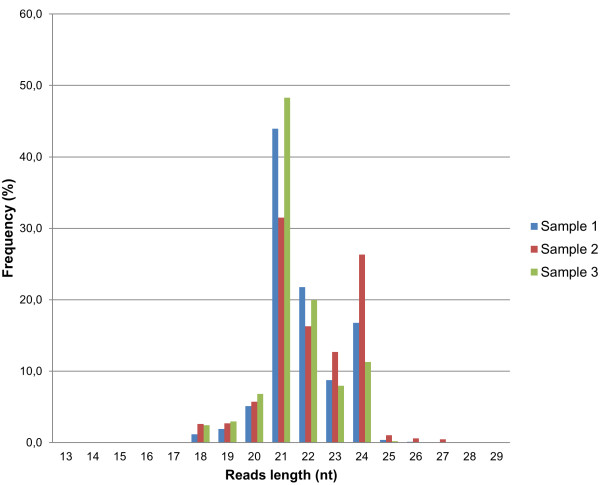
**Summary of the sequence lengths distribution.** The graphs were generated from the unique tags of all three samples. Most of the generated reads were 21 (> 31%), 22 (> 16%), 24 (> 11%) and 23 (> 7%) nucleotides long.

After Illumina sequencing of three small RNA libraries, a total of 25161201, 24037208 and 26342479 reads were generated. The removal of low-quality tags and contaminants, and further reads clustering resulted in sets of 5866438, 6139583, 5687116 unique sequences, respectively. The examination of their length distribution showed that most of the generated reads had 21 (> 31%), 22 (> 16%), 24 (> 11%) and 23 (> 7%) nucleotides (Figure 1), which are also the most frequent sizes of the known *Brassica* plant miRNAs [28,37-39]. In the next step, the *B. oleracea* sequences of 25 tRNAs, 39 rRNAs, 1 snoRNA and 64 repeat-associated RNAs were downloaded and matched to the unique tags to remove ncRNAs from the reads collection. The respective exclusion of 2054/2122/2115 rRNAs, 1350/2846/1285 tRNAs, 5/5/3 snoRNAs and 5734/5475/5155 repeat-associated RNAs reads reduced the data sets to 5857295/6129135/5678558 sequences. To eliminate mRNA degradation products, the GSS and EST sequences were first assembled with the CAP3 program. A further comparison of the generated 60950 contigs and 366969 singletons to the NR (NCBI) database provided a collection of the *B. oleracea* protein-coding sequences. The obtained data, together with 469 CDS (NCBI) sequences, served as the reference set in a BlastN search, which resulted in the removal of 7311/5406/4493 tags, respectively, having a high probability of being part of exons. The remaining reads were then used in the homology search for known *B. oleracea* miRNAs.

Due to the lack of a complete *B. oleracea* genome, the filtering steps of this bioinformatic analysis were repeated using the *B. rapa* and *A. thaliana* sequences. From the GenBank and Rfam database, 63 tRNAs, 666 rRNAs, 568 snoRNAs, 181 snRNAs and 2 scRNAs were obtained and aligned with the unannotated tags, resulting in the respective elimination of 686/1267/1267, 5374/6080/6080, 788/776/852, 2998/3697/3697 and 131/123/175 reads. The remaining tags were then searched to exclude 8014/9227/7401 sequences, showing high similarity to some repeat-associated RNAs and 221750/156305/134627 tags which were most likely part of CDS’s; thus, resulting in the final sets of 5610243/5946254/5519967 unannotated unique reads. Detailed information about the number of eliminated tags, representing different types of RNA sequences, is summarized in Table 1.

**Table 1:** Annotations of the clean reads from three cabbage samples

**Category**	Unique reads S1/S2/S3*	Total reads S1/S2/S3*	Percent (%) of total reads S1/S2/S3*
**Clean reads**	5866438 / 6139583 / 5687116	24911901 / 23542905 / 25950963	100%
**rRNAs**	7428 / 8202 / 8195	131763 / 212780 / 142748	0.53% / 0.90% / 0.55%
**tRNAs**	2035 / 4113 / 2552	21321 / 60177 / 38836	0.08% / 0.25% / 0.15%
**snRNAs**	2998 / 3697 / 3697	28552 / 23286 / 32047	0.01% / 0.09% / 0.12%
**snoRNAs**	793 / 781 / 855	1474 / 1375 / 1543	0.0059% / 0.0058% / 0.0059%
**scRNAs**	131 / 123 / 175	2322 / 1202 / 2059	0.0093% / 0.0051% / 0,0079%
**Exons**	229061 / 161711 / 139120	1075206 / 756268 / 669788	4.3% / 3.2% / 2.6%
**Repeat-associated sequences**	13748 / 14702 / 12556	85629 / 80407 / 73632	0.34% / 0.34% / 0.28%
**Unannotated reads**	5610243 / 5946254 / 5519967	23565634 / 22407410 / 24990310	94.5958% / 95.111% / 96.2862%

*Sample 1, sample 2, sample 3.

### Identification of conserved miRNAs in cabbage leaves

As mentioned in the previous paragraph, the unannotated reads were used in a BlastN search against a collection of 202 *B. oleracea* miRNAs, which were obtained from the PMRD database and the Wang et al. study [35]. Identical sequences or those with one gap/mismatch outside the “seed” region (2–8 nt at 5′ end of the molecule) were selected and clustered into known miRNA families. A similar analysis was performed for the unannotated tags that remained after filtering out the *A. thaliana* and *B. rapa* ncRNAs, exons and repeat-associated RNAs. However in this case, exactly 10588 known miRNAs from 126 different plant species (except for the *B. oleracea*) served as a reference set and only BlastN alignments with no more than 3 mismatches/gaps outside the “seed” region were selected.

Performed analysis of high-throughput sequencing data confirmed the presence of 261 miRNAs from 62 miRNA families in the cabbage leaves. After the sequence similarity search, 8 of the *B. oleracea* miRNAs that were collected in the PMRD database and 58 from 193 miRNAs candidates presented in the Wang et al. study [35] were successfully identified in all three libraries. The most likely explanation for the lack of the remaining known *B. oleracea* miRNAs in generated data sets is their absence or low expression level in the mature cabbage leaves. In addition to the mentioned *B. oleracea* molecules, homologues of the 195 known miRNAs from other plant species were discovered. Among the identified miRNA families, which most of them were shown to be abundant in plants closely related to the *Brassicaceae*, the MIR169 contained the highest number of members (29). The less numerous were the MIR167, MIR166, MIR171 and MIR396 families, represented by 22, 19, 13 and 13 members, respectively (see Additional file 1: Table S1). The relative abundances of discovered cabbage miRNAs were estimated by the mean values of the normalized number of miRNA tags in all three libraries. The following molecules had the highest abundance level: bol-miR167a (bna-miR167a homologue, 684250 reads), bol-miR166a (csi-miR66a homologue, 495299 reads), bol-miR157a (170940 reads), bol-miR168c (128281 reads), bol-miR172a (67605 reads), and bol-miR156c (29530 reads) (see Additional file 1: Table S2). The presented results are consistent with high-throughput sequencing studies of small RNAs from plants such as the *Brassica rapa*[28], *Poncrius trifoliata*[40] and *Arabidopsis thaliana*[41]. Among the sequences obtained from cabbage reads analysis, the antisense miRNAs (miRNAs*) were also present. Because miRNAs* are usually quickly degraded after miRNA:miRNA* duplex processing, these antisense sequences can be rarely found in conventional sequencing results. Their existence, however, may indicate the abundance of mature miRNAs or important functionality of both particles [42,43]. In the analyzed results, 23 miRNAs* belonging to 13 families were identified. In general, their expression levels were relatively low; nevertheless, the highest levels were shown by bol-miR162c* (13070 reads), bol-miR162b* (1184 reads), bol-miR157a* (aly-miR157a* homologue, 662 reads), bol-miR160a* (bra-miR160a* homologue, 287 reads), and the bol-miR172b* (184 reads). These molecules also represent antisense sequences of the most abundant miRNA species (see Additional file 1: Table S2).

### Discovery of novel *B. oleracea* miRNAs

To designate novel cabbage miRNAs, approximately 5530000 unannotated unique sequences were mapped to the *B. oleracea* contigs and singletons and to the *A. thaliana* and *B. rapa* genomes. The 2412302/2418729/2361432 perfectly or near perfectly matched tags were then used in the Mireap method to predict pre-miRNAs. The minimum folding energy (MFE) of the obtained 2562/1449/2475 hairpin precursors was evaluated with the UNAFold program. The remaining sequences with MFEs less than -18 kcal/mol, were analyzed by the NOVOMIR and HuntMi software to exclude pseudo pre-miRNAs and discard precursors not classified as typical for the *A. thaliana*. To further improve the quality of the predicted molecules, the selected stem-loop structures, in the final stage of described analysis, were evaluated manually. As a result of these elimination steps, 26 new potential miRNAs including 3 miRNAs*, were selected from all three libraries (see Additional file 1: Table S3).

### Cabbage trans-acting siRNA prediction

After the conserved and novel miRNAs identification, the collection of 5468000 unannotated reads together with 176631 tags representing parts of exons were subjected to the prediction of potential *B. oleracea TAS* genes. As a result of the used tool, the 202 tasiRNAs from 26 loci were proposed (see Additional file 1: Table S12). To complement the described analysis, searching for sequences homologous to the known *A. thaliana* TAS1, TAS2, TAS3 and TAS4 was performed. In this part of study, the TAS3a D7(+) was successfully identified in all three cabbage samples (mean normalized reads count: 770). The *B. oleracea* contig sequence from which mentioned TAS3a homologue originates, possess significant similarity (77%, E-value 2e^-45^) to the TAS3a loci in the *A. thaliana* (At3g17185) [44].

### Northern blot analysis of selected *B. oleracea* var. *capitata* miRNAs

**Figure 2:**
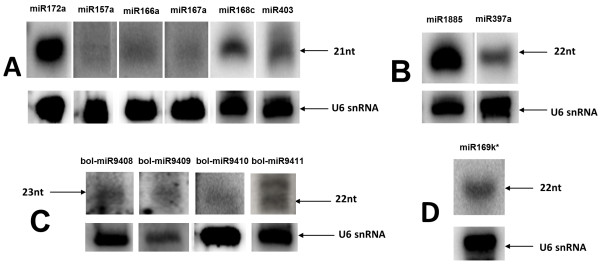
**Northern blot analysis of selected conservative and novel *B. oleracea *var. *capitata *miRNAs.** Figure represents the validation of the expression level of nine identified conservative **(A, B and D)** and four novel **(C)** miRNAs in mature cabbage leaves using the northern blot hybridization method [8]. The phosphor screens were exposured for 7 days.

After the identification of the conserved and novel cabbage miRNAs, northern hybridization experiment was conducted for 13 selected miRNA species. The sequences of the evaluated miRNAs are shown in Table 2. The presence of eight conserved miRNAs, one chosen miRNA* and four novel potential miRNAs identified in NGS give visible signals (Figure 2). The four novel, confirmed miRNAs were named by the miRBase Registry according to the standard miRNA nomenclature as the bol-miR9408, bol-miR9409, bol-miR9410 and bol-miR9411, respectively.

**Table 2:** MiRNAs, which expression have been evaluated by the northern blot hybridization method

**Lp.**	**miRNA name**	**Sequence**	**Sequence length**	**Number of reads**
**1**	bol-miR9408	GTTTCATCTTAGAGAATGTTGTC	23	58
**2**	bol-miR9409	TTTTGTTCATGACTGCATTTTC	22	218
**3**	bol-miR9410	TACTTAATTATAAGTCGTCTGG	22	1681
**4**	bol-miR9411	TACTGGACGACTTACACGGAAG	22	420
**5**	bol-miR172a	AGAAUCUUGAUGAUGCUGCAU	21	67605
**6**	bol-miR157a	UUGACAGAAGAUAGAGAGCAC	21	170940
**7**	bna-miR166a	UCGGACCAGGCUUCAUUCCCC	21	495299
**8**	bra-miR167a	UGAAGCUGCCAGCAUGAUCUA	21	684250
**9**	bol-miR168c	UCGCUUGGUGCAGGUCGGGAA	21	128281
**10**	bol-miR169k*	GGCAAGUUGUCCUUCGGCUACA	22	78
**11**	bol-miR1885	CAUCAAUGAAAGGUAUGAUUCC	22	43
**12**	bol-miR403	UUAGAUUCACGCACAAACUCG	21	2170
**13**	bol-miR397a	UCAUUGAGUGCAGCGUUGAUGU	22	1628

### MiRNA putative target prediction and annotation

**Figure 3:**
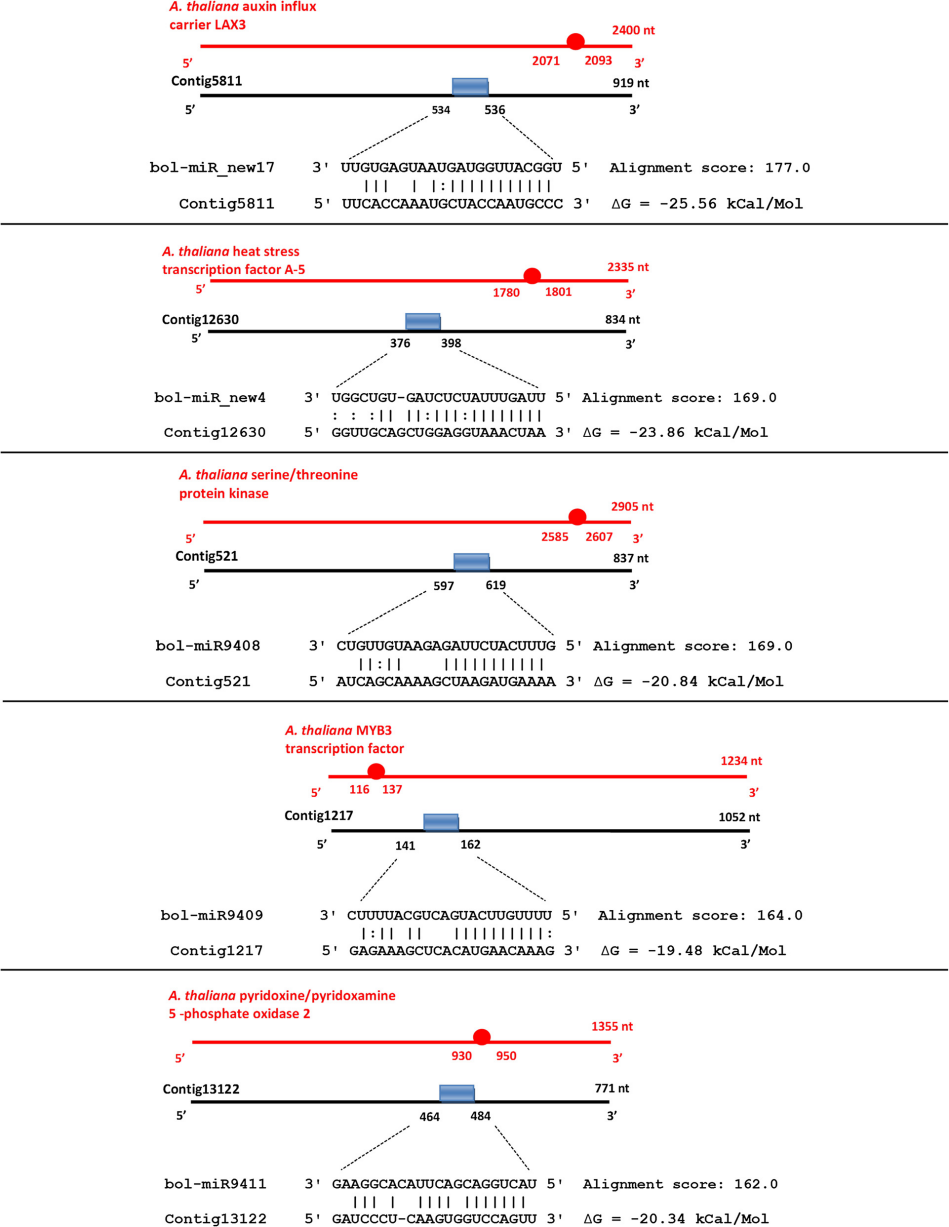
**The potential binding sites of selected targets predicted for 5 novel cabbage miRNAs.** Figure describes binding sites in the selected targets of five newly identified miRNAs: bol-miR_new4, bol-miR_new17 and also the experimentally confirmed bol-miR9408, bol-miR9409 and bol-miR9411. MiRNAs targets are represented by black line with their contig ID identifier, sequence length and annotation. Blue box indicates the miRNA binding site (exact coordinates included). The miRNA:mRNA sequence alignments, together with the calculated score and minimum free energy of the duplex, are presented beneath each target binding site. In the alignment “**|**” refers to perfect complementary between bases, “-“ represents the gap, while “:” stands for G:U wobble pair. Above each of the presented miRNAs target (contig) the scheme of the *A. thaliana* homologous cDNA sequence is placed (red line). The circle indicates the referring miRNA:mRNA binding site (exact coordinates included).

The roles of miRNAs in living organisms are associated with their sequence complementarity to specific mRNAs, which leads to the protein translation inhibition or cleavage of those mRNAs [45]. Therefore, the selection and annotation of miRNAs targets are essential steps in the designation of miRNAs functions in the cell. To better understand the importance of these identified molecules in processes that occur in cabbage leaves, miRNAs putative targets prediction was conducted. As a result of Miranda program and the rules described in “Potential targets prediction for known and novel miRNAs” section of the Methods, unique mRNAs encoding 3637 potential proteins were proposed as molecules that may interact with the identified cabbage miRNAs. The collected targets were further sorted and the best molecules with highest alignment score and lowest MFE of the structure were selected. Some of the discovered miRNAs showed high sequence complementarity to the multiple regions in one mRNA, whereas others were designated as potentially targeting a few different molecules. The potential binding sites of five novel miRNAs in their predicted selected targets are presented in Figure 3. To gather more information about the probable functions of the identified *B. oleracea* var. *capitata* miRNAs, the annotation of their potential targets was performed with the Blast2GO software. This analysis, which involved the BlastX search against the *Arabidopsis* genome, revealed many transcription and translation factors among the conserved and novel miRNAs potential targets (e.g., NAC, eIF2-γ, eIF3a, some subunits of the NF-Y complex, TCP, MYB domain protein and WRKY). In addition to these molecules, mRNAs of hormone response factor, several transporters, nucleases, kinases, ATPases, dehydrogenases, synthases, and heat shock proteins were proposed as interacting with the cabbage miRNAs (see Additional file 1: Tables S4 and S6). The group of best conserved and novel miRNAs targets were separately subjected to the GO classification and enrichment analysis, which revealed that conserved cabbage miRNAs may play essential molecular role or be involved in important biological processes, such as the metabolism (GO:0008152, adjusted P-value 7.63e^-58^), response to stimulus (GO:0050896, adjusted P-value 1.15e^-27^), cellular component organization (GO:0016043, adjusted P-value 1.30e^-20^), localization (GO:0051179, adjusted P-value 1.72e^-19^), biological regulation (GO:0065007, adjusted P-value 8.02e^-19^), transport (GO:0006810, adjusted P-value 1.62e^-16^), protein modification process (GO:0036211, adjusted P-value 3.62e^-11^), cell communication (GO:0007154, adjusted P-value 2.89e^-9^), signaling (GO:0023052, adjusted P-value 4.84e^-7^), development (GO:0009791, adjusted P-value 1.28e^-6^), and growth (GO:0040007, adjusted P-value 1.01e^-5^). The similar overrepresented terms were also found in the result of GO analysis conducted for novel miRNAs targets (see Additional file 1: Tables S8 and S10). The GO enrichment examination was additionally proposed for each individual MIR family. For some of the identified miRNAs, the statistically significant results could not be obtained; however, the calculated enrichment for the rest of analyzed MIRs showed several overrepresented GO terms, which may help specify biological roles of these individual miRNAs families. As an example, the “glucose 6-phosphate metabolic process” term was enriched in bol-miR158 annotations (GO:0051156, adjusted P-value 0.001), the “auxin binding” was overrepresented in bol-miR393 list of GO terms (GO:0010011, adjusted P-value 0.009), while the “cellular response to oxidative stress” was shown to be enriched in bol-miR_new10 annotations (GO:0034599, adjusted P-value 0.001) (see Additional file 1: Tables S9 and S11). To specify the exact processes that the selected potential target proteins may participate in, enzyme mapping on the processing pathways from the KEGG database was additionally performed. The obtained results demonstrated that many of the identified conserved and novel *B. oleracea* miRNAs may regulate starch and sucrose, purine, amino-sugar and nucleotide-sugar, glycerolipid and fatty acid metabolism, glycolysis/gluconeogenesis, flavonoid biosynthesis, carbon fixation, and oxidative phosphorylation (see Additional file 1: Tables S5 and S7). These predictions are consistent with potential targets proposals and their experimental validations from several different studies concerning the miRNAs in *Brassicaceae* plants [28,35,37,46,47]. With respect to the miRNAs functions, determining the potential occurrence of their predicted targets in the cellular components is also important. The GO classification demonstrated that the selected proteins, whose mRNAs may be targeted by conserved and novel miRNAs are located in the nucleus, plasma membrane, cytosol, vacuole, and chloroplasts.

## Discussion

At the early stage of the miRNA research field, miRNA species were discovered and analyzed individually or in small groups. Currently, the development of high-throughput sequencing and bioinformatic approaches have enabled the identification of large sets of miRNAs that occur in certain tissues, cells or at particular stages. Using the above mentioned techniques, the miRNA collections have been identified from many species, including model organisms or important food crops [28,37,40,48,49]. One of the vegetables that is significant in some countries due to its health and economic benefits is the *Brassica oleracea* var. *capitata* (cabbage). To date, there were only 9 *B. oleracea* miRNAs collected in the Plant microRNA Database and 193 *B. oleracea* miRNA candidates proposed by Wang et al. in the genome-based comparative analysis approach [35]. An Illumina small RNA sequencing was performed to identify a complete set of conserved and novel miRNAs in the mature cabbage leaves. A large number of unique reads was generated from three prepared libraries. The distribution of their lengths showed that the most abundant were 21 and 22 nt long sequences (Figure 1), which is typical for plant miRNAs. This result also agreed with the Wang et al. study [35] and was the first indication of the number of potential miRNAs in the obtained sets of tags. In fact, the raw data processing and the BlastN search stages of the bioinformatic analysis enabled the identification of 261 conserved miRNAs from 62 families.

As expected, the molecules that showed the highest abundance in cabbage leaves represents miRNAs highly conserved in plants belonging to *Brassicaceae* family, as well as in few others not closely related species. This concerns the miR167a, miR166a, miR168c, miR157a and miR172a [28,37,41,48,50,51]. In terms of the members, the MIR169, MIR166 and MIR167 families were the most numerous. Furthermore, the miRNAs representing families described only in *Brassicaceae* species [50,51] were also found in the sequencing reads (e.g., the MIR158, MIR1885, MIR838, MIR824, MIR391 and MIR1140 families). Mature miRNA sequences are generated from pre-miRNAs, which are further processed into the miRNA:miRNA* duplex. The miRNA strand retains in the system, while miRNA* molecule is degraded quickly after its release from the mentioned dsRNA. Hence, its abundance level is relatively low compared with the sense strand but it can still serve as an indicator of the mature miRNA quantity [52]. In the present study, 23 miRNAs* from 13 families were identified. The most numerous were bol-miR162c*, bol-miR162b*, bol-miR157a*, bol-160a* and bol-miR172b*, and their corresponding miRNAs representing the most abundant MIR families (see Additional file 1: Tables S1 and S2). As mentioned earlier, Wang et al. proposed 193 potential *B. oleracea* miRNAs. The occurrence of only 58 candidates was verified in this study, which might be explained by low expression level of the remaining potential miRNAs in the mature cabbage leaves or their general low expression in the whole plant. The absence of these Wang’s et al. miRNAs candidates might also suggest that they are potential organ-specific molecules, which are present only in e.g., flowers, roots, stems and seeds.

An important part of the present study was the prediction of novel miRNAs. Those reads that remained unannotated after the selection of the conserved miRNAs were subjected to further bioinformatic analysis. As a result, 26 potential new molecules were proposed. The verification of the abundance of 13 (known and novel) miRNAs in cabbage leaves was performed by northern blot hybridization, which resulted in confirming the presence of the validated molecules. The sequence length distribution analysis of the mentioned 13 miRNAs (see Additional file 2) was additional verification of these particles as a properly annotated miRNA species. With respect to the miRNA sequence length, it is worth mentioning that in some plants, the miRNA-mediated target cleavage leads to the production of secondary siRNAs that are able to silence other genes *in trans* (tasiRNAs) [53]. Moreover, this regulation depends on the presence of an asymmetric miRNA:miRNA* duplex, in which the initiatory miRNA is 22 nt rather than 21 nt long [54]. Among the newly identified miRNAs, 9 were the most abundant in the 22 nucleotide size category (see Additional file 1: Table S3). In turn, preformed tasiRNA prediction analysis proposed 202 potential tasiRNAs from 26 loci and sequence homologous to the *A. thaliana* TAS3a. Although further studies are needed, at this point some speculations can be made that the identified novel cabbage miRNAs might be part of the initial miRNA:miRNA* duplex participating in the generation of some of these secondary siRNAs.

The majority of plant miRNAs possess perfect or near-perfect complementarity to their targets [45]. This feature is used in many bioinformatic prediction approaches and enables the relatively easy selection of potential target mRNAs. At the same time, it could serve as the first step in experimental validation and as a potential source of information about the miRNA functions. To determine the roles of the identified miRNAs in cabbage leaves, putative target prediction and annotation was performed in present research. The Miranda program selected 3637 mRNA molecules potentially interacting with the conserved and novel miRNAs, from which the best targets were collected and further analyzed. Several different mRNA sequences were predicted to be targeted by the same miRNA particle, suggesting that these miRNAs may play multiple roles in cabbage. The analysis of the obtained results also showed that one mRNA is potentially targeted by a number of different miRNAs molecules, which might be an additional confirmation of the quality of this specific target prediction. The targets proposed in present study included several translation and transcription factors. A few belonged to the WRKY proteins family, which in plants plays a regulatory role in response to bacterial, fungal or viral infections, wounding, or other stresses [55]. Certain WRKY members have also been reported to regulate leaf senescence [56]. Squamosa promoter binding-like protein 4 (SPL4) is another selected transcription factor and a key player in *Arabidoposis* developmental transition from the juvenile to adult phase. Its expression has been shown to be regulated by the miR156 [57,58], which is consistent with the putative target prediction presented in this study. The MYB proteins family is one of the larger groups of transcription factors in plants. Its members function in a variety of important processes, i.e. development, metabolism, hormone signal transduction, disease resistance, and responses to the environmental stresses [59-61]. In *A. thaliana* several MYB proteins were identified and validated as targets for the miR159 [62]. Member of this family, the MYB9, has been also proposed as the best target for the cabbage miR159. The well-known plant molecules – auxins, are implicated in many aspects of plant growth and development as well. In the presence of these hormones, the F-box proteins binds to the transcriptional regulators called Aux/IAA molecules, participating at the same time in their ubiquitination and degradation [63,64]. Described receptors such as Auxin Signaling F-box Proteins (AFBs), has been demonstrated by Chen et al. [65] and predicted by miRanda method to be targeted by miR393. In addition to the above mentioned molecules, mRNA of the TPC4 transcription factor was proposed and confirmed in earlier studies to interact with the miR319. This polypeptide is responsible for the leaf growth and senescence [66,67]. Another putative target protein, which is important in plant senescence and vegetation changes, is the chlorophyllase 1. Mentioned enzyme, potentially targeted by cabbage miR399a, was reported to be involved in light controlled chlorophyll (Chl) degradation, exactly the catalytic reaction converting the Chl to carboxylic acid chlorophyllide (Chlide) and phytol [68,69]. MiRNA species not only play important roles in plants growth and development but also affects significantly their response to the various environmental conditions (e.g., micro-/macro-nutrient and water limitations) [21,70]. The stress-induced regulatory processes are associated with the change in abundance levels of the specific molecules, such as plasma membrane sulfate transporter (ST2.1) and subunits of the NF-YA transcription factor. The mRNA of the *B. oleracea* ST2.1, protein homologous to the *A. thaliana* SULTR2.1, was predicted to be the putative target for the cabbage miR395a. Overexpressed miR395 has been earlier reported to inhibit the transcription of the *SULTR2.1* gene during the sulfur deficiency controlling, therefore, the sulfate accumulation and allocation in *Arabidopsis*[71]. MiR169g and miR169n have been shown to target *NF-YA*[72], which in effect down-regulates the NF-Y transcription factor in *Triticum aestivum* drought-affected leaves [73].

To gain additional functional insights into the miRNA-target interaction and ensure the accuracy of the presented annotation study, the proposed putative targets were subjected to the GO enrichment analysis and further mapped on metabolic pathways obtained from the KEGG database. The generated list of overrepresented GO terms gave general view of the roles, which group of all novel and conserved miRNAs molecules may play in the cabbage leaves, e.g. development regulation, involvement in metabolic processes, establishment of the localization, transport, growth, and several other primary biological processes. One of the most enriched GO term was the “response to stimulus”. The transcription factors mentioned in the previous paragraph, such as subunits of NF-Y, MYB proteins, NAC-domain containing molecules, auxin response factors, and miRNAs interacting with them have been shown to be involved in plants responses to environmental conditions – salt stress, mechanical stress, water or nutrient deficiency, and etc. [21,72]. The conserved as well as the novel miRNAs presented in this study were identified in *B. oleracea* leaves, therefore their targets are expected to be involved in some organ-specific processes. For selected putative targets the “leaf development” was not found in the results of GO enrichment analysis, however, the term closely related to it – “organ development” has been shown to be overrepresented. Based on the enrichment analysis performed for targets of each individual MIR family, the assumption can be made that some of the identified miRNA groups have specific molecular function; e.g. miR393 participate in auxin binding, bol-miR_new1 play potential role in responses to various stimulus, while bol-miR_new10 is likely to be involved in cellular responses to oxidative stress. The performed KEGG pathway mapping proposed that many of identified cabbage miRNAs may take part in the same biological processes but at different stages. One of the processing networks, which include the largest number of miRNAs potential targets, was glycolysis/gluconeogenesis. As a result of this set of chemical reactions, glucose is converted into pyruvate, and at the same time, energy is released. In plants, the mentioned energy, in a form of high-energy ATP and NADH compounds, is crucial to power respiration. Moreover, the intermediates of glycolysis are used in biosynthesis pathways of other molecules, such as amino acids, fatty acids and secondary metabolites [74,75]. Glycerolipids metabolism has also been selected as a metabolic pathway that may involve many of miRNAs best putative targets enzymes. Glycerolipids are major lipids in plant chloroplasts. In addition, they are responsible for photosynthesis efficiency and plant growth under various phosphate availability conditions [76]. In addition to the pathways described above, a large group of predicted cabbage miRNA targets may participate in starch, sucrose, purine, methane, fatty acid, nitrogen, amino-sugar, and nucleotide-sugar metabolism or in the carbon fixation and citrate cycle. Mentioned biological processes are sufficient for normal plant growth and development, which at the same time implies that the regulation of these metabolic pathways by identified miRNAs is crucial for the plant to function.

## Conclusions

The presented study shows, for the first time, a set of conserved and novel miRNAs that occur in mature *B. oleracea* var. *capitata* leaves. The analyzed high-throughput sequencing data were consistent with previous reports concerning the miRNAs in *Brassicaceae* species [28,37,39,41,46] and showed, in a broader sense, the evolutionary relationship between cabbage and other plants. The potential target designation suggested the functions and roles that the identified miRNAs might play in cabbage leaves. In the future, experimental verification of some of the predicted target molecules will be important; particularly those that might be crucial for agricultural performance, such as genes responsible for abiotic stress responses (cold, heat and drought). Because the complete genome of this food crop is not known yet, the full set of cabbage miRNAs and their targets could not be designated. However, presented research does provide important and fairly extensive knowledge about mentioned molecules and their functions. Additionally, this knowledge may be useful in studies concerned with methods to increase the yield and improve the ability of cabbage to adapt to various environmental conditions.

## Methods

### RNA extraction, small RNA library preparation and sequencing

Using the modified Trizol method as described by [8], total RNA enriched with sRNA was isolated from the mature cabbage leaves (*B. oleracea* var. *capitata*, cultivar Balbro) in three independent biological replicates. Next generation sequencing (NGS) using Illumina HiSeq technology was performed at the Beijing Genomics Institute (BGI) in China, according to the manufacturer’s protocol.

### Bioinformatic analysis of small RNA tags

Sequencing reads were generated from three constructed, independent small RNA libraries. The raw data obtained for each sample were further bioinformatically analyzed to clean, remove unnecessary tags and identify sequences representing the conserved and novel miRNAs, and also the tasiRNAs. Due to the lack of the complete *B. oleracea* genome, the data processing pipeline used in this analysis was slightly different from the one commonly applied in recent high-throughput sequencing studies (see Additional file 3) [28,29]. The small RNAs sequence data discussed in present research have been deposited in the NCBI’s Gene Expression Omnibus repository under accession number GSE45578.

The first step of raw data processing concerned the removal of low quality tags, exactly the sequences with: any N bases, more than 4 bases whose quality score was lower than 10 and more than 6 bases whose quality score was lower than 13. The reads shorter than 18 nucleotides, containing 5′ primer contaminants, containing poly A tail or missing 3′ primer, and insert tags were also excluded from the data sets. The remaining tags were combined into unique reads and then lengths of their sequence were summarized. To eliminate all other small non-coding RNAs, clean tags from each sample were annotated as tRNAs, rRNAs, scRNAs, snRNAs, and snoRNAs. The sequences of these ribonucleic acids were collected from the GenBank (191.0 release) and Rfam (11.0 release) database. The similarity was investigated using the BlastN algorithm [77], allowing one gap and one mismatch in the alignment. The E-value threshold was set at 0.01. The same parameters were used to remove the repeat-associated RNAs (obtained from the Repbase v.17.11 and Plant Repeat Database http://plantrepeats.plantbiology.msu.edu/downloads.html). Because the *B. oleracea* genome is still incomplete, to avoid the inclusion of mRNA fragments in the analyzed reads, the protein coding genes had to be first selected from the available genomics sequences. To do so, the 179213 EST and 680984 GSS sequences were downloaded from the NCBI database (September 2012, http://www.ncbi.nlm.nih.gov), processed and further assembled with CAP3 software (default parameters, http://seq.cs.iastate.edu/) [78]. The generated contigs and singletons were aligned with the BlastX algorithm to the non-redundant protein database (NR), with an E-value threshold of 0.001 (NCBI, ftp://ftp.ncbi.nih.gov/blast/db/). The designated protein-coding sequences, together with several CDSs collected from NCBI (http://www.ncbi.nlm.nih.gov/nuccore), served as a reference set for the BlastN method, which was used to select and eliminate mRNA degradation products from reads of each sample. In exons fragments search stage, the E-value threshold was set at 0.01 and one gap and one mismatch were allowed in the alignment.

After removing potentially false positive tags that could interfere with the obtained results, the next step of the presented analysis was to select sequences that possess significant similarity to known *B. oleracea* miRNAs. To date, there are only 9 *B. oleracea* miRNAs collected in the miRBase database (http://www.mirbase.org/) [22] and Plant microRNA Database (PMRD, http://bioinformatics.cau.edu.cn/PMRD/) [23]. Recently, Wang et al. reported 193 *B. oleracea* miRNA candidates, identified from EST and GSS sequences via a homology search against known miRNAs [35]. In the described step of performed analysis, the BlastN algorithm with an E-value threshold of 0.01 was used to select the reads with no more than one mismatch/gap (outside the “seed” region) and with sequence coverage that differs by a maximum of two nucleotides. Tags that were not selected in this step remained unannotated. Since much of the *B. oleracea* genomic data is still missing, the reads filtering phase of the analysis was repeated with the use of the *Brassica rapa* and *Arabidopsis thaliana* sequences. The choice of those organisms was dictated by the fact that all three plants belong to the *Brassicaceae* family (i.e., share the same ancestor), with the split between the *Brassica* and *Arabidopsis* lineages being approximately 20 million years ago [79,80]. In addition, their close homology, manifested by sequence similarity and conserved colinearity of gene order and content, has been verified in many studies [81-87]. To remove tags that reveal homology to the *A. thaliana* and *B. rapa* tRNAs, rRNAs, snRNAs, snoRNAs and scRNAs, sequences of mentioned ncRNAs were collected and aligned with the unannotated reads using BlastN method. All tags that possessed less than three mismatches or gaps in the alignment and E-value did not exceed the 0.01 threshold, were removed from the data sets. A similar analysis was performed for elimination of the repeat-associated sequences and exons fragments (using *B. rapa* and *A. thaliana* data obtained from the Repbase, Plant Repeat Database, and http://www.phytozome.net/). The collection of the remaining reads was further searched to find sequences that are potential members of the conserved plant miRNA families, gathered in the Plant microRNA Database (PMRD). The BlastN method with a 0.01 E-value threshold was used. Reads with an alignment E-value below the threshold, that possessed no more than 3 mismatches/gaps (outside the “seed” region) and with their sequence coverage differing by no more than 2 nucleotides, were annotated as sequences homologous to the known plant miRNAs. MIRs which were not described in plants closely related to the *Brassicaceae* and abundance of their identified members was below 15 reads (average normalized value), were removed from final miRNA families collection. The remaining unannotated tags were further used to predict tasiRNAs and novel cabbage miRNAs.

### Prediction of novel miRNAs in cabbage leaves

The first step in the prediction of new cabbage miRNAs was mapping of the unannotated tags to the *B. oleracea* contigs and singletons (generated from EST and GSS sequences) by the SOAP v1.11 method (http://soap.genomics.org.cn/index.html) [88] – no mismatches were allowed, while the “seed” region size was set at 8. Unique tags that perfectly matched these contigs and singletons were subjected to the next step of analysis. The remaining reads were also mapped to the genomes of the *A. thaliana* and *B. rapa.* The necessary genomic sequences were available at http://www.phytozome.net/, while the mapping was conducted with the SOAP v1.11 and Bowtie 0.12.8 software (http://bowtie-bio.sourceforge.net) [89]. In both methods, the parameters were set so as to allow one mismatch in the alignment. Additionally, for the SOAP v1.11 tool the “seed” region size was set at 8. For all mapped tags, representing potential new miRNAs, the hairpin precursors were generated by the Mireap method developed by the Beijing Genomics Institute (BGI, http://sourceforge.net/projects/mireap/). Mentioned stem-loop structures were obtained only when tags were mapped to the arm region of the hairpin precursor and the free energy of the folded structure, calculated by RNAfold [90], was lower than -18 kcal/mol [91,92]. The resulting hairpin precursors were also evaluated with the default parameters of Mireap program, which used the following filters for the miRNAs candidates - structures have a minimum of 16 base pairs in the miRNA:miRNA*, the bulge size as well as the asymmetry between miRNA and miRNA* is no more than 4 nucleotides. The folding energy of the selected precursors was further additionally evaluated using UNAFold software, where -18 kcal/mol threshold was also set (http://mfold.rna.albany.edu/?q=DINAMelt/software) [93]. To obtain more reliable miRNA candidates, the precursors that remained after the previous filtering steps were subjected to analysis with the NOVOMIR (http://www.biophys.uni-duesseldorf.de/novomir/) and HuntMi method (http://adaa.polsl.pl/agudys/huntmi/huntmi.htm). The NOVOMIR tool discriminates pre-miRNAs from all other RNAs and is based on several statistical models (including HMMs) and known secondary structure prediction algorithms (RNAfold and RNAshapes). The high sensitivity and specificity of NOVOMIR was shown for the *A. thaliana* pre-miRNAs [94]. In the mentioned tool, the maximum free energy threshold for the folded structures was set at -18 kcal/mol, while the other parameters remained as default. The HuntMi is a taxon-specific approach for the miRNA hairpin classification, based on ROC-select strategy combined with the random forest method. The described software comes with the G_*m*_-optimized models for human, animals, plants (including separate *A. thaliana* model selected in this analysis) and viruses [95]. The obtained final set of the novel *B. oleracea* miRNAs was check manually according to the annotation criteria described by Meyers et al. [96]. Potential novel miRNAs were discarded from the final collection when they were reported as deriving from heterogeneous precursor positions or there was no clear dominance of their specific sequence from one arm of the proposed hairpin structure. To normalize the number of conserved and novel miRNAs the library scaling method was used [97].

### Potential *B. oleracea* trans-acting RNAs prediction

MiRNAs are required for the biogenesis of another small RNAs species, tasiRNAs. To assess whether phased 21-nt sRNA characteristic of tasiRNA loci can be designated from the obtained data sets, the TA-SI prediction tool was used (the UEA Small RNA Workbench, http://srna-workbench.cmp.uea.ac.uk/) [98]. Firstly, the mentioned method matches all sequences to the reference genome. Then, it implements the algorithm described by the Chen et al., which search for the phased 21-nt sRNA increments (potential *TAS* genes) and calculates their probability on the basis of a hypergeometric distribution [99]. In this part of performed analysis, the unannotated tags together with full collection of reads that possess significant similarity to the exons fragments, served as sRNA datasets. The *B. rapa* genome was used as a reference. The parameters of the TA-SI prediction tool were set so as to remove all tags with mapping abundance lower than 4 and discard potential *TAS* locus, which calculated P-value was below the 0.001 threshold. To identify sequences homologous to the *A. thaliana* TAS1, TAS2, TAS3 and TAS4, mentioned tasiRNAs were downloaded from the pssRNAMiner web server (http://bioinfo3.noble.org/pssRNAMiner/index.php?dowhat=Dataset) and aligned with remaining unannotated tags by the BlastN method. The E-value threshold was set at 0.001. To normalize the number of proposed tasiRNAs the library scaling method was used [97].

### Northern hybridization analysis of selected cabbage miRNAs

Thirteen of the identified conserved and novel miRNAs were chosen to validate their expression level in mature cabbage leaves using the northern blot hybridization method. Hybridization was carried out as described by Szarzynska et al. [8]. Briefly, RNA (45 μg) was resolved in 15% denaturing polyacrylamide electrophoresis (PAGE) and transferred to a Hybond-NX nylon membrane (GE Healthcare, Little Chalfont, Buckinghamshire, UK), followed by UV-crosslinking. Probes for the identification of individual miRNAs were labeled with γ^32^P-ATP (6000 Ci/mmol; Hartmann-Analytic, Braunschweig, Germany) using T4 polynucleotide kinase (Roche, Mannheim, Germany) and purified on IllustraMicroSpin G-25 Columns (GE Healthcare, Little Chalfont, Buckinghamshire, UK). A probe complementary to U6 snRNA (5′TCATCCTTGCGCAGGGGCCA-3′) was used as a loading control. Hybridization was performed overnight at 42°C. The RNA size was estimated using the ^32^P-labeled Decade Marker System (Ambion, Austin TX, USA). The membranes were washed twice with washing buffer (0.1% SDS, 2x SSC) for 40 min at 42°C. The radioactive signals were recorded using phosphor screens (Fujifilm Co., Ltd., Tokyo, Japan) and scanned using a FLA-5100 Fluorescent Image Analyzer (Fujifilm Co., Ltd., Tokyo, Japan). The generated images were further visually analyzed to find the expression signal for each of 13 miRNAs.

### Potential targets prediction for known and novel miRNAs

An important aspect of present study was the prediction of potential targets for the collected, conserved and novel cabbage miRNAs. In this part of the analysis the miRanda software was used (http://www.microrna.org/microrna/getDownloads.do) [100], which target searching procedure is based on the sequence complementarity and thermodynamic stability of the miRNA:mRNA duplex. The *B. oleracea* protein-coding EST sequences (annotated at an earlier step) and mRNAs from NCBI served as the set of potential miRNA targets. The prediction was performed with the following rules and parameters of the miRanda method: (1) G:U base pairing is permitted but scored less (score +2) than canonical base pairing (score +5), (2) alignment score threshold of 130, (3) minimum free energy of structure less than -17 kcal/mol and (4) alignment of the “seed” region should not contain any gaps or non-canonical base pairs. The targets proposed by the miRanda method were sorted according to the higher alignment score and lower MFE. Then, the top best 10–20 molecules, depending on the primary size of potential targets set, were selected. To better understand the biological roles and designate the potential processes involving these targets, the Blast2GO program was used. The GO annotations were obtained based on the BlastX search against the *A. thaliana* database (from NCBI) with an E-value threshold of 1e^-6^. The KEGG (Kyoto Encyclopedia of Genes and Genomes) and InterPro databases were also searched with an E-value of 1e^-10^. Additionally, to obtain the general functional information about the identified miRNAs, the GO terms enrichment analysis (molecular function and biological process) for their best targets was performed with the Ontologizer tool (http://compbio.charite.de/contao/index.php/ontologizer.over.html) [101]. The mentioned analysis was conducted for each of individual MIR family and separately for group of all conserved and novel miRNAs. The “Term-For-Term” algorithm with the Bonferroni correction for multiple testing was chosen in the calculations. The P-value threshold was sat at 0.05.

### Availability of supporting data

The data sets supporting the results of this article are included in articles additional files and available in the Gene Expression Omnibus (GEO) repository, GSE45578 and https://www.ncbi.nlm.nih.gov/geo/query/acc.cgi?acc=GSE45578.

## Abbreviations

miRNA: microRNA; miRNA*: miRNA star; pri-miRNA: primary miRNA; pre-miRNA: miRNA precursor; tasiRNA: *trans*-acting siRNA.

## Competing interests

We have not received, in the past five years, reimbursements, fees, funding, or salary from an organization that may in any way gain or lose financially from the publication of this manuscript, either now or in the future. Moreover, such organization is not financing this manuscript. We do not hold any stocks or shares in any organizations that may in any way gain or lose financially from the publication of this manuscript, either now or in the future. We do not hold or are currently applying for any patents relating to the content of the manuscript. We have not received reimbursements, fee, funding, or salary from an organization that holds or has applied for patents relating to the content of the manuscript. We do not have any other financial competing interest. There is also no other non-financial competing interests to declare in relation to this manuscript.

## Authors’ contributions

AL performed the bioinformatic analysis of Illumina sequencing data. ZS-K and HP designed and performed the northern blot hybridization. AL wrote the manuscript with assistance from ZS-K, HP and PZ. AL, ZS-K, LP and PZ conceived of the study, and participated in its design and coordination. All authors read and approved the final manuscript.

## Supplementary Material

Additional file 1 **This additional file contains 12 subtables.** The annotations of the tables are as follows: **Table S1.** – MiRNA families identified in *Brassica oleracea* var. *capitata* (cabbage) leaves. **Table S2.** - Identified conserved miRNAs in *Brassica oleracea* var. *capitata* (cabbage) leaves with sequence homologous to known plant miRNAs (including the known *B. oleracea* miRNAs). **Table S3.** - Predicted novel cabbage miRNAs, together with their pre-miRNA sequences and secondary structures in dot-bracket format. **Table S4.** – List of the best predicted targets for discovered conserved cabbage miRNAs. **Table S5.** – List of the designated KEGG processing pathways, in which the identified conserved *B. oleracea* miRNAs may participate in. **Table S6.** – List of the best predicted targets for potential novel cabbage miRNAs. **Table S7.** – List of the designated KEGG processing pathways, in which the identified novel *B. oleracea* miRNAs may participate in. **Table S8.** – List of the overrepresented GO terms calculated for the whole collection of conserved cabbage miRNAs best targets. **Table S9.** – List of the enriched GO terms calculated for the best targets of each individual conserved *B. oleracea* miRNA family. **Table S10.** – List of the overrepresented GO terms calculated for the whole collection of potential novel miRNAs best targets. **Table S11.** – List of the enriched GO terms calculated for the best targets of each individual novel *B. oleracea* miRNA family. **Table S12.** – Predicted cabbage tasiRNAs, together with their proposed TAS locus, chromosome location (*B. rapa* reference), strand and calculated P-value. Click here for file

Additional file 2 **Sequence length distribution of the selected conserved and novel cabbage miRNAs, which expression were evaluated by northern blot analysis.** The graphs were generated from the mean values of the normalized number of miRNA tags in all three libraries. The sequence length distributions for evaluated molecules are typical for plant miRNA species. Click here for file

Additional file 3 **Workflow of the cabbage sequencing data analysis.** The obtained raw reads were cleaned and filtered out of the *B. oleracea* ncRNAs, repeat-associated RNAs and exon fragments. The remained tags were match to the known miRNAs in order to select from obtained data sets all conserved molecules. The unannotated reads were further mapped to the *B. oleracea* contigs and singletons (generated from GSS and EST sequences). Matched tags were used to predict the miRNA precursors, which structures and energies were additionally evaluated. The remained unannotated tags together with reads representing exon fragments were subjected to the tasiRNAs prediction part of the study. In next step of the analysis, the target designation and annotation were carried out for the selected novel and known cabbage miRNAs. The filtering and mapping steps were repeated with the use of the *B. rapa* and *A. thaliana* sequences (dashed arrows). Each stage of the performed analysis is detailed described in Methods sections. Blue hexagons represent the data used and generated in the following processing steps (pink rectangles) of the analysis. Click here for file
