# Supplementary material for: High-throughput sequencing identification of novel and conserved miRNAs in the Brassica oleracea leaves
Source: BMC Genomics. 2013 Nov 19;14:801. doi: 10.1186/1471-2164-14-801 (PMC3840582; doi:10.1186/1471-2164-14-801)
Supplement: Additional file 2 — Sequence length distribution of the selected conserved and novel cabbage miRNAs, which expression were evaluated by northern blot analysis. The graphs were generated from the mean values of the normalized number of miRNA tags in all three libraries. The sequence length distributions for evaluated molecules are typical for plant miRNA species. [file 1471-2164-14-801-S2.pdf]

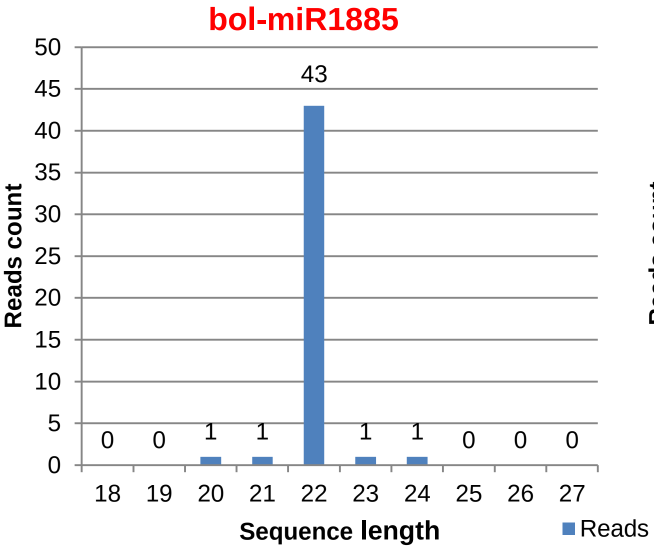
bol-miR1885
Reads count
50
45
40
35
30
25
20
15
10
5
0
0 0 1 1 43 1 1 0 0 0
18 19 20 21 22 23 24 25 26 27
Sequence length
Reads

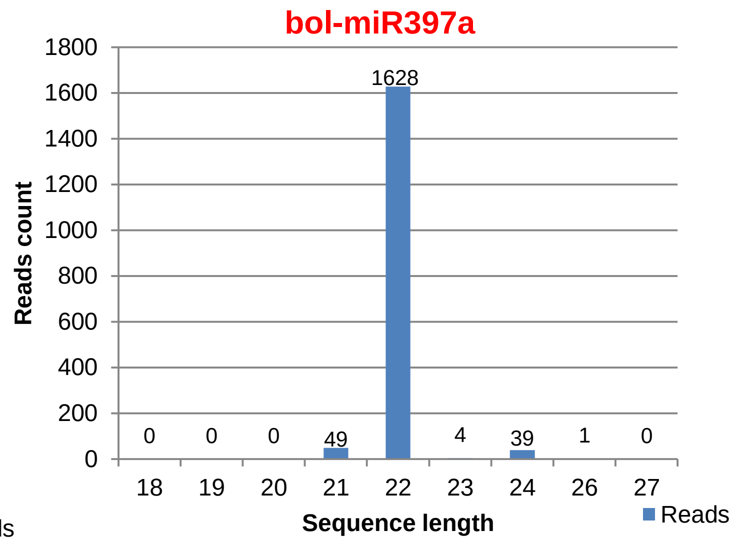
bol-miR397a
Reads count
1800
1600
1400
1200
1000
800
600
400
200
0
0 0 0 49 1628 4 39 1 0
18 19 20 21 22 23 24 26 27
Sequence length
Reads

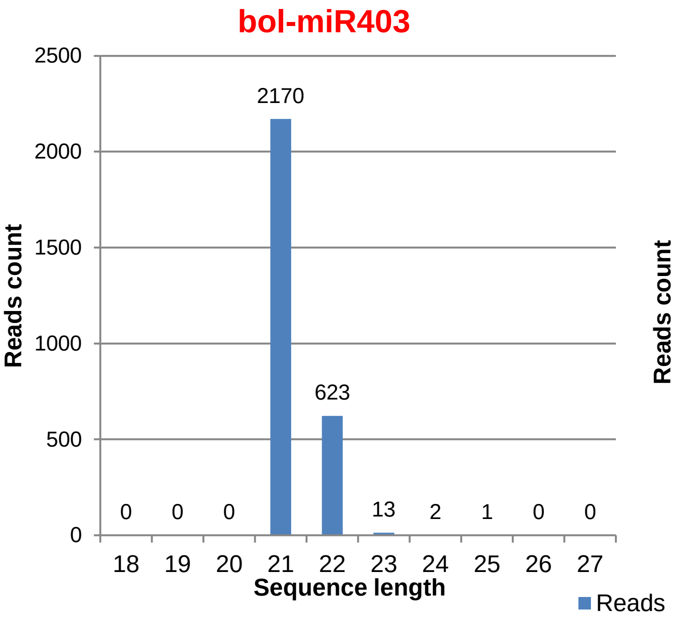
bol-miR403
Reads count
2500
2000
1500
1000
500
0
0 0 0 2170 623 13 2 1 0 0
18 19 20 21 22 23 24 25 26 27
Sequence length
Reads

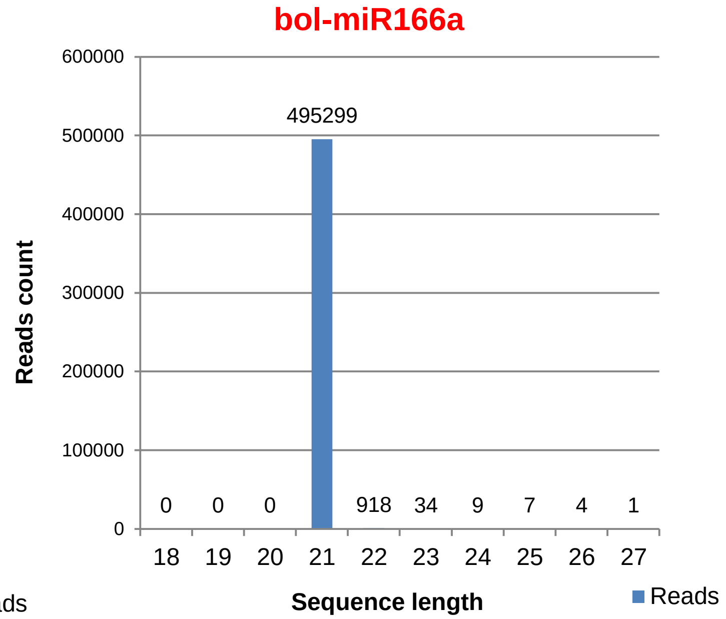
bol-miR166a
Reads count
600000
500000
400000
300000
200000
100000
0
0 0 0 495299 918 34 9 7 4 1
18 19 20 21 22 23 24 25 26 27
Sequence length
Reads

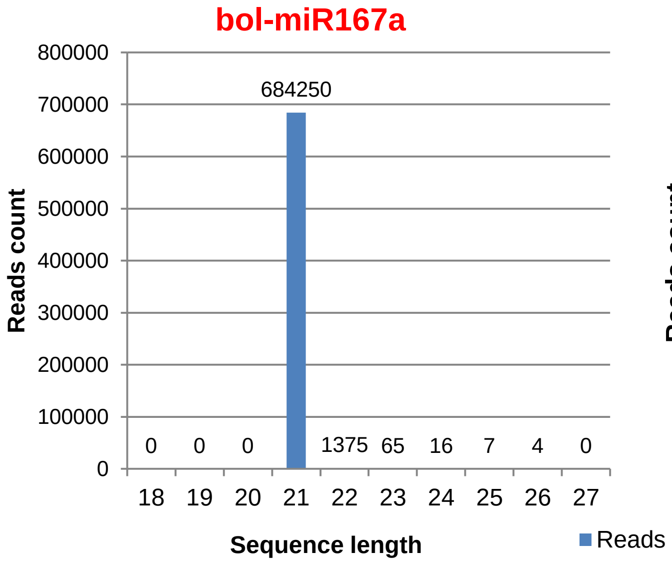
bol-miR167a
Reads count
800000
700000
600000
500000
400000
300000
200000
100000
0
0 0 0 684250 1375 65 16 7 4 0
18 19 20 21 22 23 24 25 26 27
Sequence length
Reads

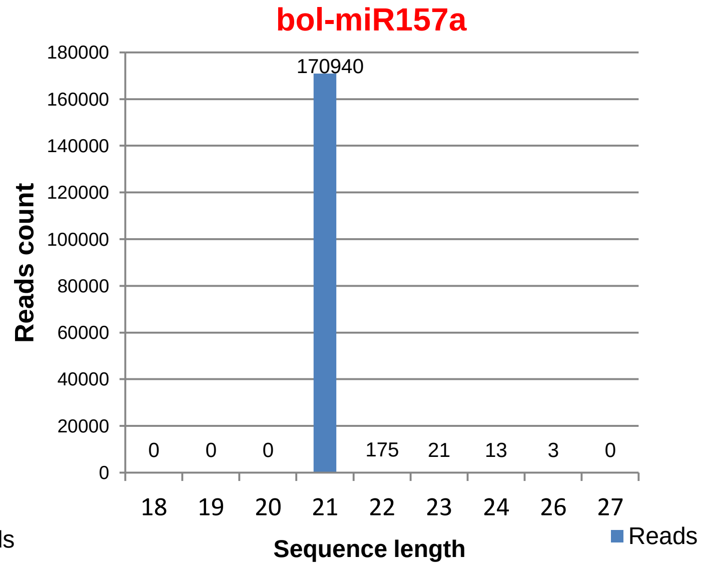
bol-miR157a
Reads count
180000
160000
140000
120000
100000
80000
60000
40000
20000
0
0 0 0 170940 175 21 13 3 0
18 19 20 21 22 23 24 26 27
Sequence length
Reads

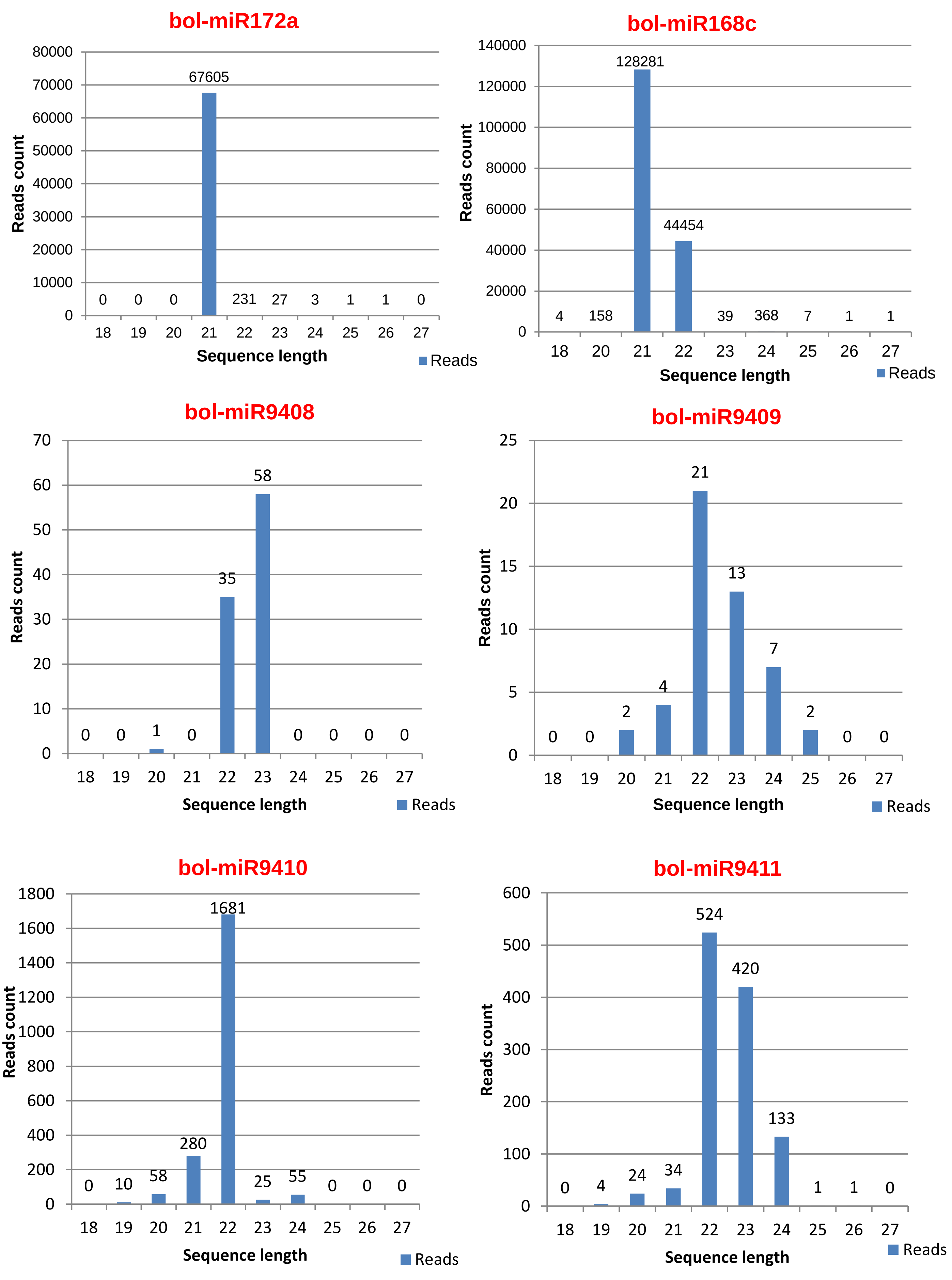
bol-miR172a
Reads count
Sequence length
67605
0 0 0 231 27 3 1 1 0
18 19 20 21 22 23 24 25 26 27
Reads
bol-miR168c
Reads count
Sequence length
128281
44454
4 158 39 368 7 1 1
18 20 21 22 23 24 25 26 27
Reads
bol-miR9408
Reads count
Sequence length
0 0 1 0 35 58 0 0 0 0
18 19 20 21 22 23 24 25 26 27
Reads
bol-miR9409
Reads count
Sequence length
0 0 2 4 21 13 7 2 0 0
18 19 20 21 22 23 24 25 26 27
Reads
bol-miR9410
Reads count
Sequence length
0 10 58 280 1681 25 55 0 0 0
18 19 20 21 22 23 24 25 26 27
Reads
bol-miR9411
Reads count
Sequence length
0 4 24 34 524 420 133 1 1 0
18 19 20 21 22 23 24 25 26 27
Reads

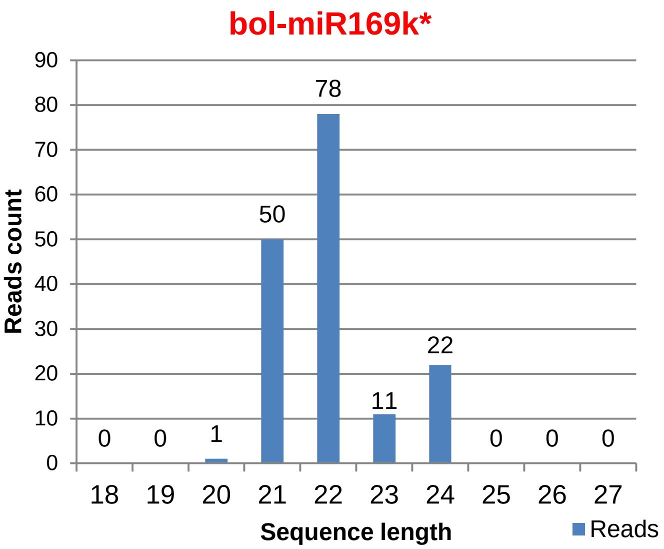
bol-miR169k*
Reads count
90
80
70
60
50
40
30
20
10
0
0
0
1
50
78
11
22
0
0
0
18
19
20
21
22
23
24
25
26
27
Sequence length
Reads
