# Supplementary figures and images for: High-throughput sequencing identification of novel and conserved miRNAs in the Brassica oleracea leaves
Source: BMC Genomics. 2013 Nov 19;14:801. doi: 10.1186/1471-2164-14-801 (PMC3840582; doi:10.1186/1471-2164-14-801)

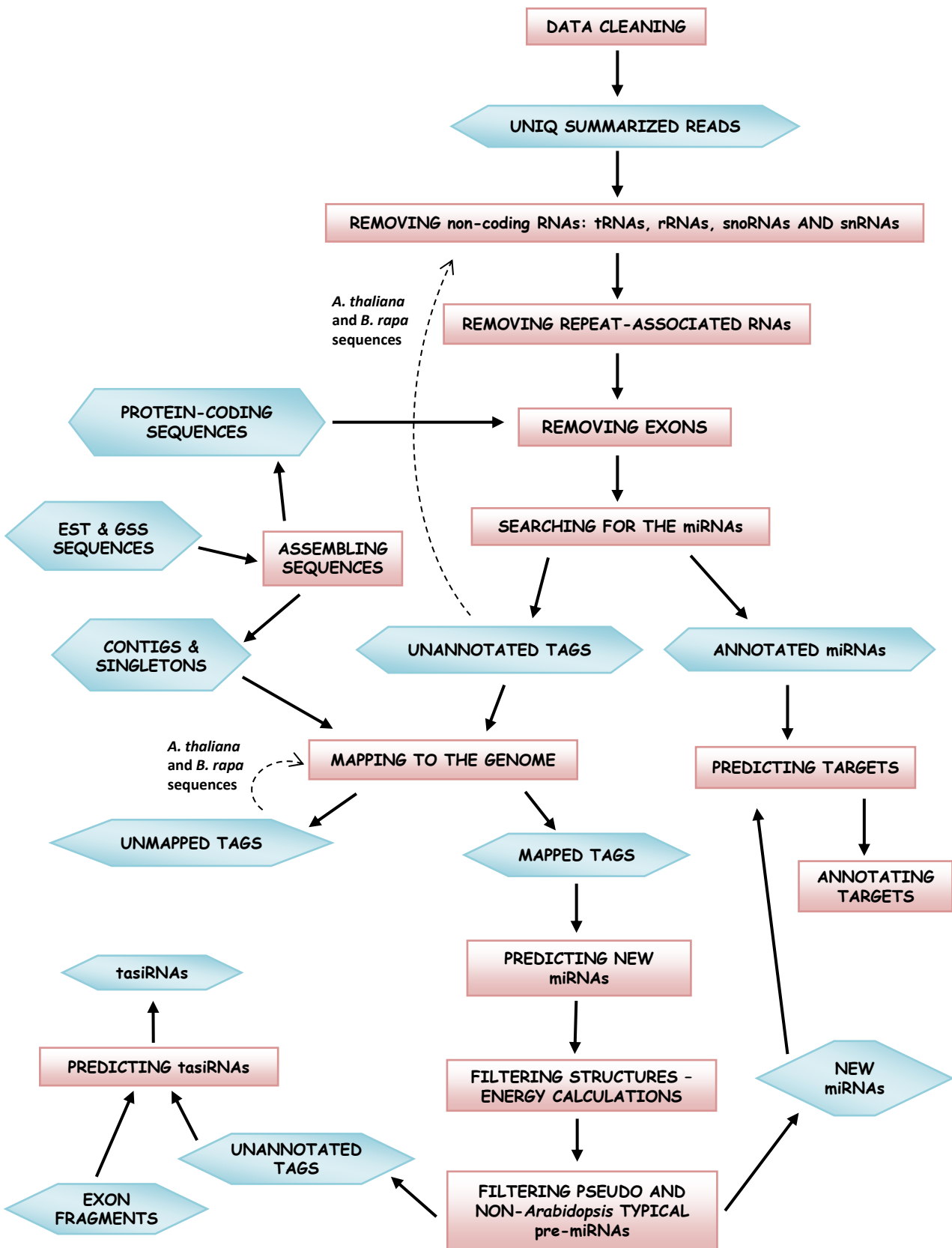

Supplement: Additional file 3 — Workflow of the cabbage sequencing data analysis. The obtained raw reads were cleaned and filtered out of the B. oleracea ncRNAs, repeat-associated RNAs and exon fragments. The remained tags were match to the known miRNAs in order to select from obtained data sets all conserved molecules. The unannotated reads were further mapped to the B. oleracea contigs and singletons (generated from GSS and EST sequences). Matched tags were used to predict the miRNA precursors, which structures and energies were additionally evaluated. The remained unannotated tags together with reads representing exon fragments were subjected to the tasiRNAs prediction part of the study. In next step of the analysis, the target designation and annotation were carried out for the selected novel and known cabbage miRNAs. The filtering and mapping steps were repeated with the use of the B. rapa and A. thaliana sequences (dashed arrows). Each stage of the performed analysis is detailed described in Methods sections. Blue hexagons represent the data used and generated in the following processing steps (pink rectangles) of the analysis. [file 1471-2164-14-801-S3.pdf]
